# Evolution of Transmissible Gastroenteritis Virus (TGEV): A Codon Usage Perspective

**DOI:** 10.3390/ijms21217898

**Published:** 2020-10-24

**Authors:** Saipeng Cheng, Huiguang Wu, Zhenhai Chen

**Affiliations:** 1 College of Veterinary Medicine, Yangzhou University, Yangzhou 225009, China; mohacsp@163.com; 2 Joint International Research Laboratory of Agriculture and Agri-Product Safety, The Ministry of Education of China, Yangzhou University, Yangzhou 225009, China; 3 Institute of Comparative Medicine, Yangzhou University, Yangzhou 225009, China; 4 Jiangsu Co-Innovation Center for Prevention and Control of Important Animal Infectious Diseases and Zoonoses, Yangzhou University, Yangzhou 225009, China

**Keywords:** transmissible gastroenteritis virus, phylogeny, codon usage bias, mutation pressure, selection pressure

## Abstract

Transmissible gastroenteritis virus (TGEV) is a coronavirus associated with diarrhea and high mortality in piglets. To gain insight into the evolution and adaptation of TGEV, a comprehensive analysis of phylogeny and codon usage bias was performed. The phylogenetic analyses of maximum likelihood and Bayesian inference displayed two distinct genotypes: genotypes I and II, and genotype I was classified into subtypes Ia and Ib. The compositional properties revealed that the coding sequence contained a higher number of A/U nucleotides than G/C nucleotides, and that the synonymous codon third position was A/U-enriched. The principal component analysis based on the values of relative synonymous codon usage (RSCU) showed the genotype-specific codon usage patterns. The effective number of codons (ENC) indicated moderate codon usage bias in the TGEV genome. Dinucleotide analysis showed that CpA and UpG were over-represented and CpG was under-represented in the coding sequence of the TGEV genome. The analyses of Parity Rule 2 plot, ENC-plot, and neutrality plot displayed that natural selection was the dominant evolutionary driving force in shaping codon usage preference in genotypes Ia and II. In addition, natural selection played a major role, while mutation pressure had a minor role in driving the codon usage bias in genotype Ib. The codon adaptation index (CAI), relative codon deoptimization index (RCDI), and similarity index (SiD) analyses suggested that genotype I might be more adaptive to pigs than genotype II. Current findings contribute to understanding the evolution and adaptation of TGEV.

## 1. Introduction

Transmissible gastroenteritis virus (TGEV) is a porcine enteropathogenic coronavirus, which causes watery diarrhea, severe villous atrophy, and high mortality in piglets. Pigs of different ages can be infected by TGEV and newborn piglets, under two weeks of age, are the most susceptible [1]. In adult pigs and piglets greater than 3 weeks of age, the response to TGEV is milder, causing loss of appetite and diarrhea for 1 to 2 days [2]. TGEV was first reported in the United States in 1946 [3], and it was subsequently identified in Europe, Asia, Africa, and South America, causing heavy losses in the global pig breeding industry [4,5,6,7,8,9,10].

TGEV belongs to the *Alphacoronavirus 1* species, *Alphacoronavirus* genus, *Orthocoronavirinae* subfamily, *Coronaviridae* family in the order *Nidovirales* [11]. TGEV contains a single-stranded, positive-sense RNA genome with a length of approximately 28.5 kb. The TGEV genome consists of a 5′ untranslated region (UTR), *open reading frame 1a/1b* (*ORF1a/1b*), *spike* (*S*), *ORF3a*, *ORF3b*, *envelope* (E), *membrane* (*M*), *nucleocapsid* (*N*), *ORF7*, and 3′ UTR [12,13,14]. S glycoprotein is related to receptor binding and cellular fusion [15,16]. It is the major target of neutralizing antibodies and the main determinant of host cell tropism and pathogenicity [17,18,19]. *ORF1a* and *ORF1b* together encode viral replicase [13] and *ORF3a* is related to the TGEV virulence [2]. *ORF7* encodes a small hydrophobic protein, which plays an important role in the process of membrane integrity in viral replication and/or virion assembly [20]. Previous studies have indicated that TGEV can be divided into traditional and variant genotypes [21].

Generally, degeneracy or redundancy of the genetic code allows that 61 triplet codons encode all 20 amino acids except for methionine and tryptophan. The multiple codons that encode the same amino acid are termed synonymous codons. It is known that different organisms use synonymous codons with different frequencies in their coding sequences (CDSs); this is called codon usage bias. The evolution of codon usage bias is very complex. The degree of codon usage bias is affected by many factors, including mutation bias, translational selection, and dinucleotide bias [22]. Compared with the genomes of prokaryotes and eukaryotes, viral genomes have specific characteristics, for example, relying on their host cell machinery for genome replication and protein synthesis. The relationships of codon usage between viruses and their hosts affect gene expression [23], viral protein translation [24], viral virulence [25], and evasion from host’s immune system [26]. In brief, a holistic analysis of codon usage bias is essential to understanding of viral evolution, host adaptability, and genome characteristics. Herein, a wide range of bioinformatic methods was used to investigate the phylogeny, codon usage pattern, factors driving codon usage bias of TGEV, and virus adaptation toward the host. The information obtained in the study not only can provide an insight into the TGEV evolution, but also can be used to rationally design the attenuated TGEV strain that may have vaccine potential.

## 2. Results

### 2.1. Distinct Genotypes

**Figure 1:**
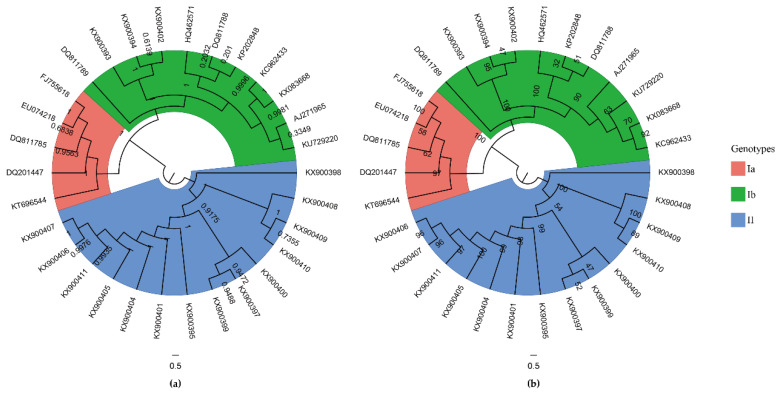
Phylogenetic trees of the complete coding sequence (CDS) of TGEV. (**a**) Bayesian inference tree of the complete CDS of TGEV. Posterior probability values calculated by MrBayes are shown at each node. (**b**) Maximum likelihood tree of the complete CDS of TGEV. Bootstrap support values computed by RAxML are indicated on the nodes of tree. Scale bar at the bottom of the figure indicates a length corresponding to 0.5 nucleotide substitutions per site. The colored sectors represent three genotypes of TGEV.

A total of 32 complete genomes of TGEV strains were downloaded from NCBI in July 2020 (Table S1). In our study, TGEV AHHF strain and TGEV/USA/Illinois139/2006 strain were identified as potential recombinants (Figure S1). After removing two recombinant sequences, 30 complete genomes of TGEV strains were further analyzed. Both the maximum likelihood (ML) tree and Bayesian inference (BI) tree showed three well-supported individual clades: genotypes Ia, Ib, and II (Figure 1). Pairwise *p*-distances between genotypes I and II, genotypes Ia and II, genotypes Ib and II, and sub-genotypes Ia and Ib were 0.0341 ± 0.0010, 0.0310 ± 0.0009, 0.0356 ± 0.0010, and 0.0127 ± 0.0006, respectively.

Considering that partial *spike* gene sequence (first 1383 nt) was more available than TGEV complete genome [21], the ML and BI trees were constructed to further study the evolutionary relationship between TGEV strains. Phylogenetic trees of partial *spike* genes (*n* = 58) showed three main clades—including genotypes Ia, Ib, and II—which were consistent with the genotyping results of TGEV complete genome (Figure S2).

### 2.2. Nucleotide U Is the Most Frequent in the TGEV Coding Sequence

Nucleotide U was most abundant (0.331 ± 0.002) in the CDS of TGEV, followed by A (0.293 ± 0.001), G (0.207 ± 0), and C (0.168 ± 0.001) (Table 1 and Table S2). The average nucleotides AU and GC contents were 0.625 ± 0.001 and 0.375 ± 0.001, respectively. The analysis of GC content at different codon positions (GC1s, GC2s, GC12s, and GC3s) revealed that the mean of GC1 (0.461 ± 0.001) was higher than that of GC12 (0.416 ± 0.001), GC2 (0.371 ± 0.001), and GC3 (0.294 ± 0.002). The nucleotides at the third positions of codons (A3, G3, U3, and C3) showed nucleotide U3 as the most abundant (0.457 ± 0.004), followed by A3 (0.249 ± 0.003), which had the highest value after U3, and then C3 (0.158 ± 0.004) and G3 (0.136 ± 0.002), indicating that A/U-end codons were enriched in the TGEV coding sequences. The average value of effective number of codons (ENC) was 44.827 ± 0.095 (<45), suggesting a moderate codon usage bias in the TGEV genome.

In order to investigate whether the genome features are similar across the entire TGEV genome, we analyzed the nucleotide contents and properties of *ORF1ab* and *spike*, which sequences account for most of TGEV complete CDS. Results showed the patterns of nucleotide contents and genome characteristics of *ORF1ab* and *spike* were similar to the patterns observed in the TGEV complete CDS (Table S3).

### 2.3. Genotype-Specific Codon Usage Pattern

**Figure 2:**
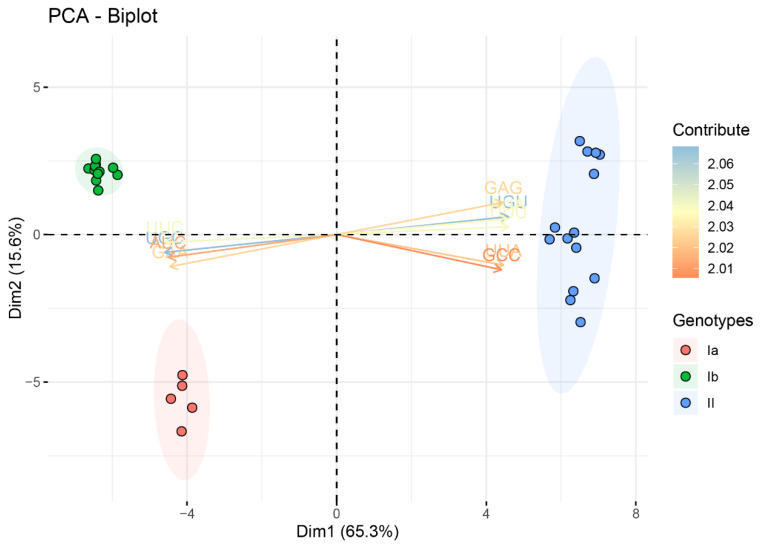
Principal components analysis (PCA) based on the RSCU values of the TGEV complete coding sequences. The significant separation in codon usage bias between the three genotypes of TGEV was present. Genotypes Ia, Ib, and II are represented in orange, green, and blue, respectively.

Relative synonymous codon usage (RSCU) analysis was performed to investigate the trend of codon usage and to further understand why A/U nucleotides are preferentially used at the third position of the codon. The preferred codons for 18 amino acids were commonly shared by three genotypes of the TGEV strains (Table 2). Surprisingly, all 18 of the preferred codons were A/U-end, including 4 A-end preferred codons (AGA[Arg], CAA[Gln], GAA[Glu], and AAA[Lys]) and 14 U-end preferred codons (GCU[Ala], AAU[Asn], GAU[Asp], UGU[Cys], GGU[Gly], CAU[His], AUU[Ile], CUU[Leu], UUU[Phe], CCU[Pro], UCU[Ser], ACU[Thr], UAU[Tyr], and GUU[Val]). Among 59 codons, about half of the codons were over-represented (RSCU > 1.6) or under-represented (RSCU < 0.6), including 12 over-represented (RSCU > 1.6) codons (20.34% of total 59 codons) and 21 under-represented (RSCU < 0.6) codons (35.59% of total 59 codons). Remarkably, the preferred codons and over-represented codons were A/U-end, while the under-represented codons were mostly G/C-ended codons except two A-end codons. The similar RSCU patterns were observed between *ORF1ab*, *spike*, and TGEV complete CDS (Table S4). In the principal component analysis (PCA) plot, the first two principal components accounted for 65.3% and 15.6% of total RSCU variations, respectively (Figure 2, Figures S3 and S4). The TGEV strains were significantly clustered into three groups: genotypes Ia, Ib, and II, which is consistent with the phylogenetic relationship of TGEV strains identified by the ML and BI analyses. These results displayed that a genotype-specific codon usage pattern was present in the TGEV strains.

### 2.4. Dinucleotides Influence the Codon Usage Pattern of TGEV

**Figure 3:**
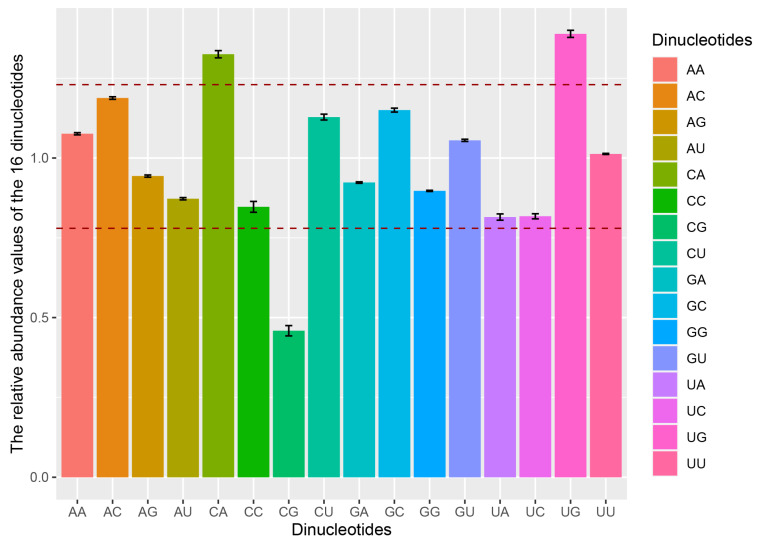
Relative dinucleotide abundance of the complete CDS of TGEV. The above and below dashed lines represent 1.23 and 0.78, respectively.

In the CDSs of *ORF1ab*, *spike*, and the TGEV genome, dinucleotides CpA and UpG were over-represented (*P*_xy_ > 1.23), whereas dinucleotide CpG was under-represented (*P*_xy_ < 0.78) (Figure 3 and Tables S5 and S6). Specially, dinucleotide ApC (*P*_xy_ = 1.349 ± 0.012) was over-represented in the *spike* gene, and dinucleotides UpA (*P*_xy_ = 0.775 ± 0.01) and GpA (*P*_xy_ = 0.778 ± 0.008) were under-represented in the *ORF1ab* and *spike*, respectively (Table S6). To investigate the possible effects of these three dinucleotides on codon usage bias, the RSCU values of CpA-containing, UpG-containing, and CpG-containing codons were analyzed. Among eight CpA-containing codons, CAA [Gln] and CAU [His] were preferentially used, and CCA [Pro] and ACA [Thr] were over-represented. Of the five codons using dinucleotide UpG, UGU [Cys], and CUG [Leu] were preferred and under-represented, respectively. Of the eight codons containing dinucleotide CpG, seven codons (GCG [Ala], CGA [Arg], CGC [Arg], CGG [Arg], CCG [Pro], UCG [Ser], and ACG [Thr]) were under-represented in the genome CDSs of TGEV. Taken together, these data suggest that the CpA over-representation and CpG depletion markedly influenced the codon usage pattern of the TGEV genome.

### 2.5. Effect of Mutation Pressure and Natural Selection on Codon Usage Bias

**Figure 4:**
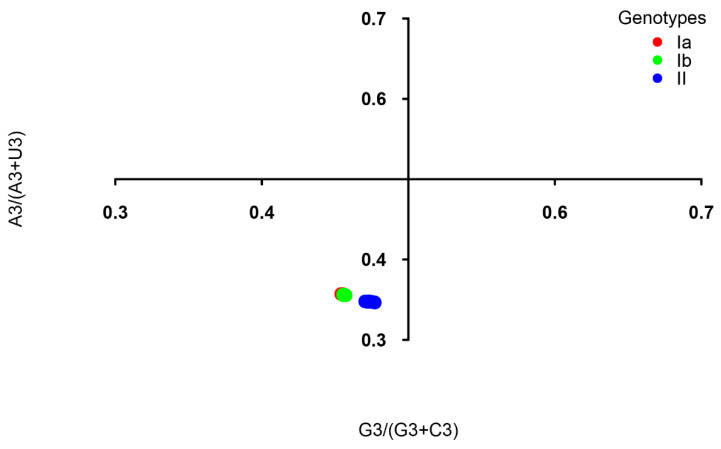
PR2 plot analysis of the TGEV complete CDS. Genotypes Ia, Ib, and II are represented in orange, green, and blue, respectively. G3/(G3 + C3) and A3/(A3 + U3) are horizontal and vertical axes, respectively.

**Figure 5:**
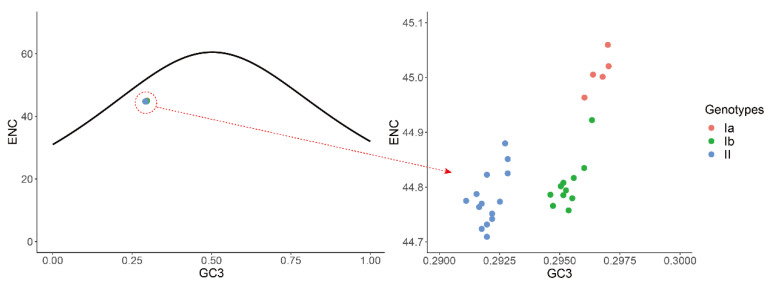
ENC plot analysis of the TGEV complete CDS. The relationships between ENC values and GC contents at the third codon position (GC3s) of synonymous codons are represent. The expected curve represents the expected ENC values toward all GC3 compositions. Genotypes Ia, Ib, and II are represented in orange, green, and blue, respectively.

**Figure 6:**
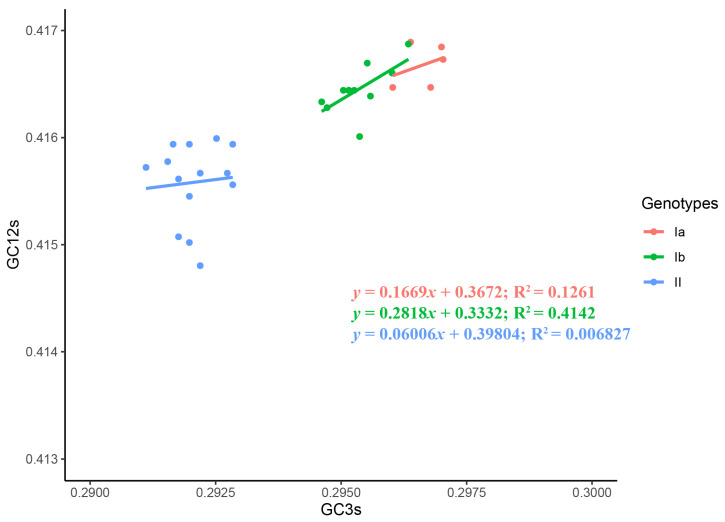
Neutrality analysis of the TGEV complete CDS. The correlation between GC12 and GC3 are represent. Genotypes Ia, Ib, and II are represented in orange, green, and blue, respectively.

To investigate the force governing codon usage patterns of TGEV, analyses of Parity rule 2 (PR2) plot, ENC plot, and neutrality were carried out. The PR2 plot showed AU-bias and GC-bias at the third codon position (Figure 4), suggesting that both mutation and selection contribute to the codon usage bias of TGEV genomes. In the ENC-GC3 plot analysis, the points representing all of the TGEV strains were below the standard curve (Figure 5), indicating that, except for mutation pressure, other factors like natural selection play a major role in the codon usage bias of TGEV. Neutrality analysis revealed no significant correlation between GC12 and GC3 in genotypes Ia (R^2^ = 0.1261, *p* = 0.5576) and II (R^2^ = 0.006827, *p* = 0.7789), suggesting that natural selection totally drives the codon usage bias of genotypes Ia and II (Figure 6). In addition, a significant positive correlation was observed between GC12 and GC in genotype Ib (*y* = 0.2818*x* + 0.3332; R^2^ = 0.4142, *p* = 0.0326), showing that the influences of mutation pressure and natural selection on codon usage bias in genotype Ib were 28.18% and 71.82%, respectively.

### 2.6. Differences in Adaptation of Genotypes toward the Host

**Figure 7:**
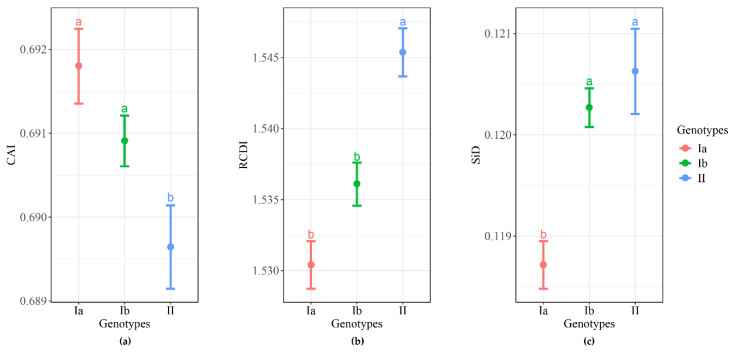
Analysis of TGEV adaptation to pig. (**a**) Codon adaptation index (CAI) of the TGEV complete CDS to pig. (**b**) Codon deoptimization index (RCDI) of the TGEV complete CDS to pig. (**c**) Similarity index (SiD) of the TGEV complete CDS to pig. Different lowercase letters indicate significant difference at the 5% level. Genotypes Ia, Ib, and II are represented in orange, green, and blue, respectively.

We explored the potential adaptation of the three clades to the host (pig). As shown in Figure 7, the mean codon adaptation index (CAI) values of genotypes Ia (0.6918 ± 0.0004) and Ib (0.6909 ± 0.0003) to the pig were significantly higher compared with the CAI value of genotype II (0.6896 ± 0.0005) (Figure 7). The relative codon deoptimization index (RCDI) analysis showed the mean RCDI value of genotype II (1.5454 ± 0.0017) to the pig was significantly higher as compared to genotypes Ia (1.5304 ± 0.0017) and Ib (1.5361 ± 0.0015) (Figure 7). The results of similarity index (SiD) analysis revealed that the mean SiD value of genotype Ia (0.1187 ± 0.0002) was statistically significantly lower compared with genotypes Ib (0.1203 ± 0.0002) and II (0.1206 ± 0.0004) (Figure 7). These results suggest that genotype I strains might be more adapted to their host (*Sus scrofa*) than genotype II strains.

## 3. Discussion

Phylogenetic analysis of the TGEV genomes revealed three distinct genotypes that are different than the previously reported traditional and variant lineages [21]. Analyses in the current study yielded a more accurate inference of the phylogeny because more methods were used and incomplete and recombinant genome sequences were excluded. Additionally, codon usage patterns of TGEV were categorized into three distinct groups based on the PCA of RSCU values, which were consistent with the well-supported genotypes Ia, Ib, and II of TGEV.

Analysis of nucleotide composition showed that nucleotide U was the most abundant in the CDS of the TGEV genome, and that the coding region and the third codon position of the TGEV genomes were A/U rich, but G/C poor, which is consistent with what has been found in porcine deltacoronavirus (PDCoV) [27] and porcine epidemic diarrhea virus (PEDV) [28] and in severe acute respiratory syndrome coronavirus 2 (SARS-CoV-2) [29]. TGEV genomes exhibited a moderate degree of codon usage bias (ENC = 44.827 ± 0.095) compared with PDCoV (52.63 ± 0.253) [27], PEDV (52.58 ± 11.24) [28], SARS-CoV-2 (48.54 ± 2.34) [30], Middle East respiratory syndrome coronavirus (MERS-CoV) (49.816 ± 0.08) [31], bovine coronavirus (BCoV) (43.78 ± 0.07) [32], and avian coronavirus infectious bronchitis virus (42.79 ± 2.25) [33]. ENC is usually used as an indicator of codon preference which is predominantly caused by under-/over-represented codons [34]. As evident from the RSCU analysis, more than half of the 59 codons were under-/over-represented in the TGEV genome. Specifically, it was noted that the preferred and over-represented codons of the TGEV genome were A/U-ended, while the most under-represented codons of the TGEV genome were G/C-ended. Overall, the peculiar compositional constraints (U and A in this case) may be the cause of codon usage bias in TGEV.

In the dinucleotide frequencies analysis of the TGEV genome, dinucleotides CpA and UpG were found to be over-represented, while the dinucleotide CpG was under-represented. The low-abundance of CpG is a genomic characteristic of positive-strand RNA viruses [35,36,37]. While over-representations of CpA and UpG are considered to be a consequence of CpG deficiency in virus genomes [37]. Notably, a prevailing influence of dinucleotides CpA and CpG on the codon usage of TGEV genome was observed, suggesting that dinucleotide bias influences codon bias of TGEV coding sequences.

To test whether GC content, nucleotide/dinucleotide frequencies, and codon usage bias were similar over the whole genome, a comprehensive comparison of these indexes of *ORF1ab*, *spike*, and TGEV complete CDS was performed. The similar patterns of the nucleotide contents, genomic properties, relative dinucleotide abundance, and RSCU were observed among *ORF1ab*, *spike*, and TGEV complete CDS. Besides, the over-represented ApC of *spike*, the under-represented UpA of *ORF1ab*, and the under-represented GpA of *spike* indicated the dinucleotide usage patterns were specific to a certain extent in the *ORF1ab* and *spike*.

Natural selection and mutation pressure are thought to be the two main factors driving codon usage patterns. Based on the results of the PR2 bias, ENC-GC3 plot, and neutrality analysis, the present study found that the codon biases of genotypes Ia and II were totally affected by natural selection, which is consistent with PEDV [28]. However, natural selection (71.82%) had a greater effect on codon usage than mutation pressure (28.18%) in genotype Ib of TGEV, which is consistent with PDCoV [27], MERS-CoV [38], SARSCoV [38], and SARS-CoV-2 [29,38]. These findings indicate that evolutionary forces driving the codon usage patterns in three genotypes of TGEV are different.

The CAI value is used to evaluate the adaptation of viral genes to the host [39]. In the CAI analysis of TGEV, genotypes Ia and Ib had higher CAI values than genotype II, suggesting a higher efficiency of protein expression in the host with genotypes Ia and Ib. Analyses of RCDI and SiD were also performed to further assess the adaptation of TGEV to the host pig. The RCDI was lower in genotypes Ia and Ib than in genotype II. Low RCDI values indicate better adaptation to the host, which is consistent with the high CAI values of genotypes Ia and Ib to the pig. Genotypes Ib and II had higher SiD values than genotype Ia, indicating that the pig might have induced stronger selection pressure on the CDS of genotypes Ib and II. The CAI, RCDI, and SiD analyses suggest that genotypes Ia and Ib might be more adapted to the host pig. These findings are consistent with a previous hypothesis that genotype II is significantly attenuated compared to genotype I of TGEV [21]. Further studies are needed to investigate the correlation between the translational efficiency, adaptation, and virulence in TGEV.

In summary, phylogenetic analysis revealed three distinct clades of TGEV strains. To our knowledge, these analyses are the first ones that reveal a moderate, but genotype-specific codon usage bias in the TGEV genome. Nucleotides (U and A) and dinucleotides (CpA and CpG) influence the codon preference of the TGEV genome. The codon usage bias of genotypes Ia and II is mainly affected by natural selection, whereas natural selection and mutational pressure emerged as a major and minor contributing factor for codon usage bias of genotype Ib, respectively. Theoretically, genotype I—including sub-genotypes Ia and Ib—may be more adapted to the pig than genotype II. Overall, this study provides insights for understanding the codon usage pattern and host adaptability of TGEV.

## 4. Materials and Methods

### 4.1. Sequence Data

The nucleotide sequences and features of the TGEV were obtained from those available up to July 2020 in the nucleotide database of National Center for Biotechnology Information (NCBI) (https://www.ncbi.nlm.nih.gov/nucleotide/). To produce high-quality genome annotations for a set of TGEV genome sequences, the CDSs for the nine genes (*ORF1a*, *ORF1b*, *S*, *nsp3a*, *nsp3b*, *E*, *M*, *N*, and *nsp7*) of TGEV were manually curated. Only the genome sequences with the complete CDSs of nine genes were retained for further analysis. The CDS of each TGEV genome was concatenated into a single super CDS with the following order: *ORF1a*-*ORF1b*-*S*-*nsp3a*-*nsp3b*-*E*-*M*-*N*-*nsp7*. The whole genome (*n* = 32), *ORF1ab* gene (*n* = 32), *spike* gene (*n* = 53), and the partial *spike* gene (first 1383 nt) (*n* = 58) used in this study were manually curated. The detailed sequence information—including accession number, strain name, location, and isolation year—are displayed in Supplementary Materials (Table S1).

### 4.2. Recombination Detection and Phylogenetic Analysis

To avoid the influence of recombination in shaping the phylogenetic tree, recombination detections for a total of 32 TGEV genome sequences were carried out. The CDSs were aligned with MACSE (version 2.03) [40]. The possible recombination events of TGEV were detected using the Recombination Detection Program (RDP, version 4.100) [41] with the default settings. Seven detection methods—including RDP, GENECONV, BootScan, MaxChi, Chimaera, SiScan, and 3Seq—were implemented in the RDP software (version 4.100) [41]. In order to avoid false positives, only results supported (Bonferroni-corrected *p* < 1 × 10^−6^) by at least four different methods were considered to be credible recombination events. After removing the recombinant sequences from the dataset, alignment and recombination detection was repeated for the remaining genome sequences until there were no recombination signals.

Non-recombinant genome sequences and the partial *spike* gene sequences (first 1383 nt) were aligned with MACSE (version 2.03) [40] using the BLOSUM62 scoring matrix, respectively. The jModelTest (version 2.1.10) [42] was used to estimate the appropriate nucleotide substitution model based on the corrected Akaike’s information criterion (AICc). The general time-reversible (GTR) model with gamma-distributed evolutionary rates (G) and invariable sites (I) (GTR + G + I) was chosen as the best fitting evolutionary model of TGEV genome evolution, while GTR + G model was identified as the best-fit model of partial *spike* gene evolution. Phylogenetic inferences were performed by maximum likelihood (ML) implemented in RAxML (version 8.2.12) [43], and by Bayesian inference (BI) implemented in MrBayes (version 3.2.7a) [44]. In the ML analysis, the node support was assessed by performing a 10,000 rapid bootstrap analysis. In the BI analysis, the Markov chain Monte Carlo (MCMC) search was conducted for 10,000,000 generations, and four chains (one cold, and three heated) sampling was carried out every 1000 generations. Tracer (version 1.7) [45] was used to check the trace files and ensure that the chains had reached convergence, which was assessed from the effective sample size (ESS) after a 10% burn-in. The first 25% of trees was discarded as burn-in and the posterior probabilities were estimated for each node. The phylogenetic trees were viewed in FigTree (version 1.4.4) (http://tree.bio.ed.ac.uk/software/figtree/). Nucleotide pairwise *p*-distances between three sister clades were calculated using MEGA X [46].

### 4.3. Compositional Properties and Principal Parameters Analysis

The contents of four constituent mononucleotides (A, U, C, and G), GC contents at the first (GC1s), second (GC2s), third (GC3s) codon positions, mean contents (GC12s) of GC1s and GC2s, and the contents of nucleotide compositions at the 3rd codon positions (A3, T3, G3, C3) were calculated using the Seqinr package (version 3.6-1) [47] of R (version 3.6.2) [48].

### 4.4. Relative Synonymous Codon Usage

RSCU quantifies the relative usage of a synonymous codon excluding the influence of amino acid composition and sequence length [49]. The RSCU values of codons in each TGEV strains were calculated according to the formula below (1) [50]

(1) $$RSCU=\frac{g_{ij}}{\sum_{j}^{ni}g_{ij}}n_{i}$$

where *g_ij_* is the observed number of the *i*_th_ codon for *j*_th_ amino acid, which has *n_i_* type of synonymous codons. The synonymous codon with an RSCU value more than 1.0 indicates a positive codon usage bias, while an RSCU value < 1.0 indicates a negative codon usage bias. A codon with an RSCU value = 1.0 indicates that the codon was chosen equally and randomly. A codon with an RSCU value > 1.6 (or <0.6) is considered to be have an over-represented (or under-represented) relative abundance compared with a random association of codon [51]. The RSCU values of 59 codons (excluding AUG[Met], UGG[Trp], and three stop codons) were calculated using the Seqinr package (version 3.6-1) [47] of R (version 3.6.2) [48].

### 4.5. Principal Component Analysis

PCA is a multivariate statistical method that reduces the dimensionality and extracts a feature from original data [52]. To determine the trends of codon usage among the different TGEV strains, PCA using a 30 × 59 matrix constructed with 59-dimensional vectors of RSCU values was carried out for each codon and 30 rows of TGEV strains. PCA was done using the Factoextra package (version 1.0.6) [53] of R (version 3.6.2) [48].

### 4.6. Relative Dinucleotide Abundance Analysis

Relative dinucleotide abundance is defined as the ratio of observed to expected dinucleotide frequency. The relative abundances of 16 dinucleotides were calculated as (2)

(2) $$P_{xy}=\frac{f_{xy}}{f_{x}f_{y}}$$

where *f_x_*, *f_y_*, and *f_xy_* represent the frequency of nucleotide *X*, the frequency of nucleotide *Y*, and the observed frequency of the dinucleotide *XY*, respectively. When *P_xy_* > 1.23 (or <0.78), the dinucleotide *XY* is considered to be over-represented (or under-represented).

### 4.7. Effective Number of Codons Analysis

ENC is used to measure the degree of deviation between codon usage and random selection. The value range of ENC is between 20 and 61 [34]. The smaller the ENC value, the stronger the codon usage bias. The ENC values were calculated as (3)

(3) $$\mathrm{ENC}=2+\frac{9}{{\bar{F}}_{2}}+\frac{1}{{\bar{F}}_{3}}+\frac{5}{{\bar{F}}_{4}}+\frac{3}{{\bar{F}}_{6}}$$

where *F_i_* (*i* = 2, 3, 4, 6) represents the average of *F_i_* in the *i*-fold degenerate codon family. The value of *F_i_* was calculated using the formula (4)

(4) $${\bar{F}}_{i}=\frac{n\sum_{j=1}^{i}{\left(\frac{n_{j}}{n}\right)}^{2}-1}{n-1}$$

where *n* is the observed number of used codons; *i* is the number of synonymous codons; and *n_j_* is the observed number of *j*_th_ codon. The ENC values were calculated using the coRdon package (version 1.4.0) [54] of R (version 3.6.2) [48].

### 4.8. ENC-Plot Analysis

ENC-plot describes the relationship between GC3 values and ENC values and provides a method to investigate the factors affecting codon bias. The expected ENC values represent the expected codon usage, which is only influenced by GC3 values. The expected ENC value was calculated according to the formula (5)

(5) $$ENC_{expected}=2+s+\frac{29}{s^{2}+{\left(1-s\right)}^{2}}$$

where *s* is the frequency of GC3.

If the point representing the observed ENC value lies on the expected curve, this indicates that the codon usage of sequence is only influenced by mutation pressure. However, if the point falls below the expected curve, this means that the codon usage bias is influenced by natural pressure.

### 4.9. Parity Rule 2 Analysis

Parity rule 2 (PR2) analysis is used to evaluate the possible function of mutation and selection on codon usage. In the PR2 plot, the GC-bias [G3/(G3 + C3)] at the third codon position and the AU-bias [A3/(A3 + U3)] at the third codon position is the abscissa and ordinate, respectively. The origin point (0.5, 0.5) indicates no bias between the influence of the mutation and selection [55,56].

### 4.10. Neutrality Analysis

Neutral analysis is used to quantitatively measure the impact of mutation and selection on codon usage bias [57]. In the neutrality plot, GC3 and GC12 are used as the horizontal coordinate and the vertical coordinate, respectively. The GC3 and GC12 contents of the genome CDSs of TGEV strains were plotted to create a scatterplot, and the relation between GC3 and GC12 was determined by the fitted regression line using R (version 3.6.2) [48]. In the neutrality analysis, if the slope of regression line is statistically significant (the closer the slope is to 1), the influence of mutation on codon usage bias is greater [57]. If the slope = 0 or the slope is not statistically significant, the codon usage bias is totally determined by natural selection [57].

### 4.11. Codon Adaptation Index Analysis

To evaluate the adaptation of the genome CDSs of TGEV strains to host, CAI was calculated using the CAIcal (version 1.4) [58]. The range of CAI is between 0 and 1. Theoretically, the higher the CAI of a sequence, the better the adaptability to the host [39]. The codon usage table of the pig (*Sus scrofa*) was obtained from the Codon and Codon Pair Usage Tables (CoCoPUTs) database [59].

### 4.12. Relative Codon Deoptimization Index Analysis

RCDI can be used to compare the similarity of codon usage between virus and host. An RCDI close to 1 indicates a better adaptation of the virus to the host [24], whereas an RCDI higher than 1 indicates that the virus is less adaptable to the host [60]. RCDIs of the TGEV sequences were calculated using the CAIcal (version 1.4) [58].

### 4.13. Similarity Index Analysis

SiD was constructed to estimate the effects of the host on the codon usage of the pathogen [61] and was calculated as (6) and (7) [62]

(6) $$R\left(A,B\right)=\frac{\sum_{i=1}^{59}a_{i}\times b_{i}}{\sqrt{\sum_{i=1}^{59}a_{i}^{2}\times \sum_{i=1}^{59}b_{i}^{2}}}$$

(7) $$D\left(A,B\right)=\frac{1-R\left(A,B\right)}{2}$$

where *a_i_* is defined as the RSCU value of the synonymous codon of the virus coding sequence and *b_i_* represents the RSCU value of the same codon of the host. *D* (*A*, *B*) is the SiD value, which represents the potential influence of the host on the virus. The higher the SiD value, the greater the influence of the host on the virus codon.

### 4.14. Statistical Analysis

Since the values of CAI, RCDI, and SiD were not normally distributed and the variances of the three genotypes were unequal, the non-parametric Kruskal–Wallis test followed by Dunn multiple comparison posthoc analysis were used. *p*-values of multiple comparisons were corrected using the Bonferroni method. A *p*-value < 0.05 was used as the cut-off criterion for statistical significance. The statistical analysis was performed using the dunn.test (version 1.3.5) [63] of R (version 3.6.2) [48].

## Figures and Tables

**Table 1:** Nucleotide contents and properties of the TGEV complete CDS

Categories	Ia	Ib	II	All
A	0.294 ± 0	0.294 ± 0	0.292 ± 0	0.293 ± 0.001
C	0.17 ± 0	0.169 ± 0	0.167 ± 0	0.168 ± 0.001
G	0.207 ± 0	0.207 ± 0	0.208 ± 0	0.207 ± 0
U	0.329 ± 0	0.33 ± 0	0.333 ± 0	0.331 ± 0.002
A3	0.251 ± 0	0.251 ± 0	0.246 ± 0	0.249 ± 0.003
C3	0.162 ± 0	0.161 ± 0	0.154 ± 0.001	0.158 ± 0.004
G3	0.135 ± 0	0.135 ± 0	0.138 ± 0	0.136 ± 0.002
U3	0.452 ± 0	0.454 ± 0	0.462 ± 0.001	0.457 ± 0.004
GC	0.377 ± 0	0.376 ± 0	0.374 ± 0	0.375 ± 0.001
AU	0.623 ± 0	0.624 ± 0	0.626 ± 0	0.625 ± 0.001
GC1	0.461 ± 0	0.462 ± 0	0.46 ± 0	0.461 ± 0.001
GC2	0.372 ± 0	0.371 ± 0	0.371 ± 0	0.371 ± 0.001
GC12	0.417 ± 0	0.416 ± 0	0.416 ± 0	0.416 ± 0.001
GC3	0.297 ± 0	0.295 ± 0.001	0.292 ± 0.001	0.294 ± 0.002
ENC	45.01 ± 0.035	44.805 ± 0.045	44.779 ± 0.05	44.827 ± 0.095

**Table 2:** Synonymous codon usage of the TGEV complete CDS. The RSCU values of 59 synonymous codons are presented. The preferred, over-represented (RSCU > 1.6), and under-represented (RSCU < 0.6) codons are displayed in bold, italics, and underscore, respectively.

Codon (Amino Acid)	Ia	Ib	II	All
GCA (Ala)	1.299 ± 0.008	1.337 ± 0.005	1.267 ± 0.013	1.298 ± 0.034
GCC (Ala)	0.458 ± 0.006	0.396 ± 0.005	0.508 ± 0.009	0.459 ± 0.052
GCG (Ala)	0.154 ± 0.005	0.136 ± 0.003	0.164 ± 0.016	0.152 ± 0.017
GCU (Ala)	***2.089 ± 0.003***	***2.13 ± 0.006***	***2.061 ± 0.014***	***2.091 ± 0.034***
AGA (Arg)	***2.745 ± 0.01***	***2.78 ± 0.01***	***2.738 ± 0.022***	***2.755 ± 0.026***
AGG (Arg)	0.914 ± 0.002	0.873 ± 0.011	0.915 ± 0.013	0.899 ± 0.023
CGA (Arg)	0.209 ± 0.008	0.208 ± 0.008	0.222 ± 0.019	0.215 ± 0.016
CGC (Arg)	0.537 ± 0.009	0.491 ± 0.009	0.54 ± 0.012	0.522 ± 0.025
CGG (Arg)	0.209 ± 0.008	0.21 ± 0.005	0.232 ± 0.009	0.22 ± 0.014
CGU (Arg)	1.387 ± 0.008	1.438 ± 0.01	1.353 ± 0.017	1.39 ± 0.041
AAC (Asn)	0.699 ± 0.005	0.694 ± 0.003	0.669 ± 0.005	0.683 ± 0.014
AAU (Asn)	**1.301 ± 0.005**	**1.306 ± 0.003**	**1.331 ± 0.005**	**1.317 ± 0.014**
GAC (Asp)	0.68 ± 0.007	0.681 ± 0.005	0.64 ± 0.009	0.662 ± 0.022
GAU (Asp)	**1.32 ± 0.007**	**1.319 ± 0.005**	**1.36 ± 0.009**	**1.338 ± 0.022**
UGC (Cys)	0.64 ± 0.002	0.638 ± 0.002	0.549 ± 0.007	0.597 ± 0.046
UGU (Cys)	**1.36 ± 0.002**	**1.362 ± 0.002**	**1.451 ± 0.007**	**1.403 ± 0.046**
CAA (Gln)	**1.229 ± 0.003**	**1.268 ± 0.004**	**1.26 ± 0.011**	**1.258 ± 0.016**
CAG (Gln)	0.771 ± 0.003	0.732 ± 0.004	0.74 ± 0.011	0.742 ± 0.016
GAA (Glu)	**1.485 ± 0.002**	**1.477 ± 0.004**	**1.425 ± 0.009**	**1.454 ± 0.029**
GAG (Glu)	0.515 ± 0.002	0.523 ± 0.004	0.575 ± 0.009	0.546 ± 0.029
GGA (Gly)	0.739 ± 0.005	0.716 ± 0.003	0.705 ± 0.01	0.714 ± 0.014
GGC (Gly)	0.582 ± 0.005	0.573 ± 0.004	0.554 ± 0.013	0.566 ± 0.015
GGG (Gly)	0.116 ± 0	0.136 ± 0.002	0.154 ± 0.007	0.141 ± 0.015
GGU (Gly)	***2.564 ± 0.01***	***2.575 ± 0.006***	***2.587 ± 0.017***	***2.579 ± 0.016***
CAC (His)	0.51 ± 0	0.499 ± 0.007	0.452 ± 0.006	0.479 ± 0.027
CAU (His)	**1.49 ± 0**	**1.501 ± 0.007**	**1.548 ± 0.006**	**1.521 ± 0.027**
AUA (Ile)	0.691 ± 0.006	0.693 ± 0.003	0.759 ± 0.007	0.723 ± 0.034
AUC (Ile)	0.49 ± 0.007	0.488 ± 0.004	0.421 ± 0.011	0.457 ± 0.035
AUU (Ile)	***1.819 ± 0.009***	***1.82 ± 0.003***	***1.821 ± 0.009***	***1.82 ± 0.008***
CUA (Leu)	0.538 ± 0.008	0.562 ± 0.005	0.512 ± 0.01	0.535 ± 0.025
CUC (Leu)	0.555 ± 0.006	0.534 ± 0.005	0.552 ± 0.01	0.546 ± 0.012
CUG (Leu)	0.258 ± 0.009	0.283 ± 0.005	0.251 ± 0.007	0.264 ± 0.017
CUU (Leu)	***2.147 ± 0.007***	***2.187 ± 0.004***	***2.155 ± 0.01***	***2.166 ± 0.018***
UUA (Leu)	1.263 ± 0.007	1.224 ± 0.006	1.315 ± 0.012	1.273 ± 0.043
UUG (Leu)	1.239 ± 0.005	1.208 ± 0.003	1.216 ± 0.011	1.217 ± 0.013
AAA (Lys)	**1.249 ± 0.002**	**1.221 ± 0.004**	**1.205 ± 0.005**	**1.218 ± 0.016**
AAG (Lys)	0.751 ± 0.002	0.779 ± 0.004	0.795 ± 0.005	0.782 ± 0.016
UUC (Phe)	0.576 ± 0.003	0.584 ± 0.003	0.517 ± 0.008	0.551 ± 0.033
UUU (Phe)	**1.424 ± 0.003**	**1.416 ± 0.003**	**1.483 ± 0.008**	**1.449 ± 0.033**
CCA (Pro)	*1.636 ± 0.005*	*1.64 ± 0.01*	*1.627 ± 0.007*	*1.633 ± 0.01*
CCC (Pro)	0.274 ± 0.011	0.247 ± 0.004	0.274 ± 0.011	0.264 ± 0.016
CCG (Pro)	0.168 ± 0	0.197 ± 0.008	0.234 ± 0.011	0.209 ± 0.027
CCU (Pro)	***1.921 ± 0.012***	***1.916 ± 0.005***	***1.865 ± 0.014***	***1.893 ± 0.029***
AGC (Ser)	0.646 ± 0.004	0.665 ± 0.007	0.614 ± 0.008	0.638 ± 0.025
AGU (Ser)	*1.624 ± 0.011*	*1.598 ± 0.004*	*1.648 ± 0.005*	*1.626 ± 0.024*
UCA (Ser)	1.295 ± 0.011	1.299 ± 0.01	1.26 ± 0.011	1.28 ± 0.022
UCC (Ser)	0.457 ± 0.001	0.44 ± 0.006	0.455 ± 0.013	0.45 ± 0.012
UCG (Ser)	0.118 ± 0.005	0.123 ± 0.004	0.15 ± 0.009	0.135 ± 0.016
UCU (Ser)	***1.861 ± 0.006***	***1.874 ± 0.014***	***1.873 ± 0.02***	***1.871 ± 0.017***
ACA (Thr)	*1.606 ± 0.016*	*1.633 ± 0.006*	1.564 ± 0.005	1.596 ± 0.033
ACC (Thr)	0.4 ± 0.003	0.414 ± 0.005	0.392 ± 0.008	0.401 ± 0.012
ACG (Thr)	0.29 ± 0.008	0.257 ± 0.003	0.313 ± 0.009	0.288 ± 0.027
ACU (Thr)	***1.704 ± 0.007***	***1.696 ± 0.005***	***1.732 ± 0.009***	***1.714 ± 0.018***
UAC (Tyr)	0.772 ± 0.003	0.781 ± 0.004	0.728 ± 0.007	0.755 ± 0.026
UAU (Tyr)	**1.228 ± 0.003**	**1.219 ± 0.004**	**1.272 ± 0.007**	**1.245 ± 0.026**
GUA (Val)	0.785 ± 0.003	0.774 ± 0.005	0.744 ± 0.005	0.762 ± 0.018
GUC (Val)	0.652 ± 0.005	0.666 ± 0.005	0.631 ± 0.012	0.648 ± 0.019
GUG (Val)	0.651 ± 0.005	0.652 ± 0.003	0.65 ± 0.004	0.651 ± 0.004
GUU (Val)	***1.911 ± 0.008***	***1.907 ± 0.003***	***1.975 ± 0.013***	***1.94 ± 0.035***

## Supplementary Materials

Supplementary Materials can be found at https://www.mdpi.com/1422-0067/21/21/7898/s1. Figure S1: Recombination analysis of TGEV strains. Figure S2: Phylogenetic trees of the partial *spike* gene of TGEV. Figure S3: PCA scree plot based on the RSCU values of TGEV complete CDS. Figure S4: The variable correlation plots of PCA. Table S1: The detailed information of TGEV strains used in this study. Table S2: The nucleotide contents and properties of TGEV complete CDS. Table S3: Comparison of nucleotide contents and properties of the *ORF1ab* gene, *spike* gene, and TGEV genome complete CDS. Table S4: Comparison of synonymous codon usage of the *ORF1ab* gene, *spike* gene, and TGEV genome complete CDS. Table S5: Relative dinucleotide abundance of TGEV complete CDS. Table S6: Comparison of relative dinucleotide abundance of the *ORF1ab* gene, *spike* gene, and TGEV genome complete CDS.
