# Supplementary material for: Evolution of Transmissible Gastroenteritis Virus (TGEV): A Codon Usage Perspective
Source: Int J Mol Sci. 2020 Oct 24;21(21):7898. doi: 10.3390/ijms21217898 (PMC7660598; doi:10.3390/ijms21217898)
Supplement: Supplementary file 1 [file ijms-21-07898-s001.zip › Supplementary Files/Supplementary_Figures.docx]

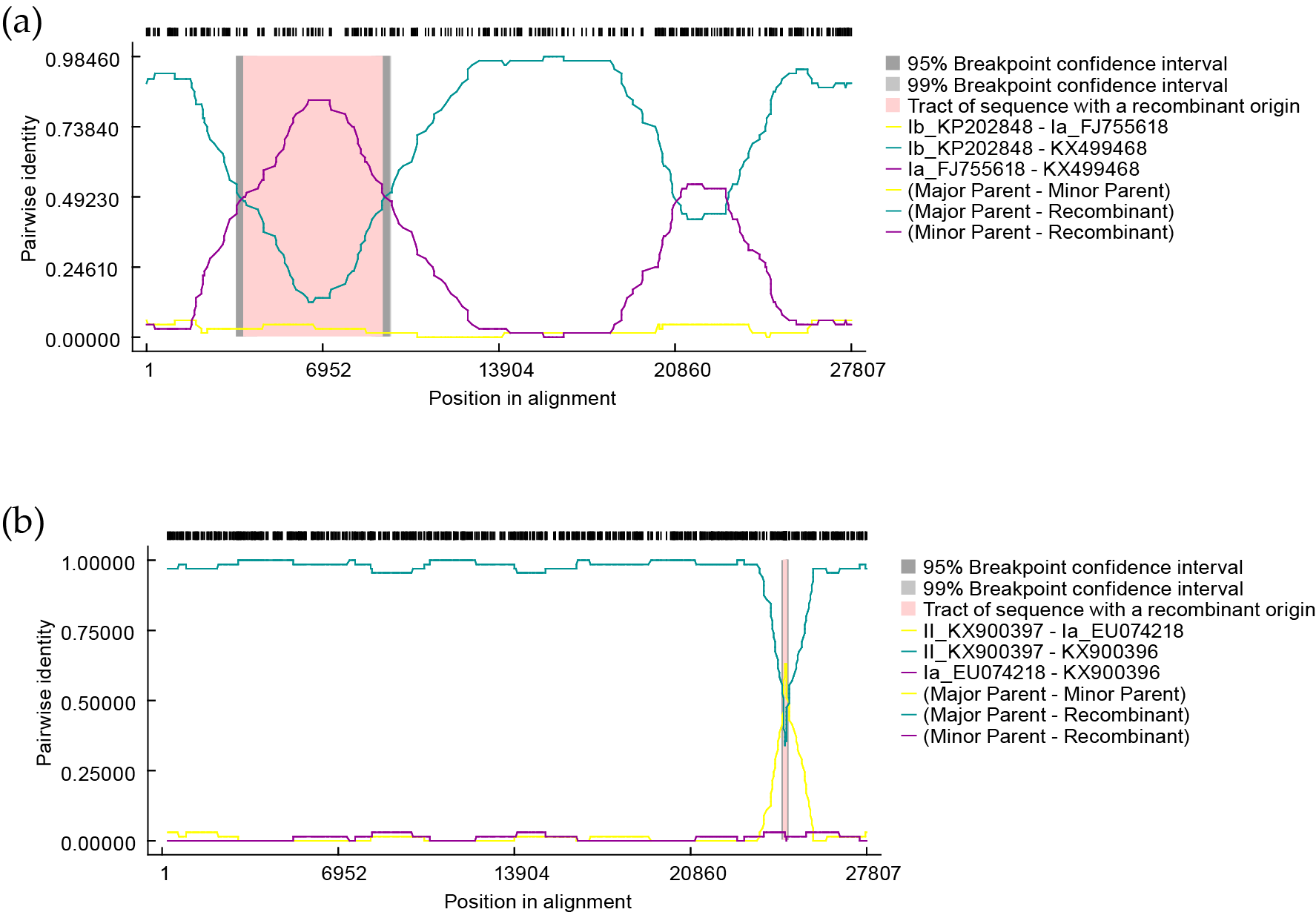


**Figure S1.** Recombination analysis of TGEV strains. **(a)** Recombination of AHHF strain. The mosaic genomic structure of the TGEV AHHF strain might originate from H16 strain and SHXB strain, which belonged to genotype Ia and Ib, respectively. **(b)** Recombination of TGEV/USA/Illinois139/2006. TGEV/USA/Illinois139/2006 strain might be recombined from attenuated H strain and TGEV/USA/NorthCarolina140/2007 strain, which belonged to genotype Ia and II, respectively.

**
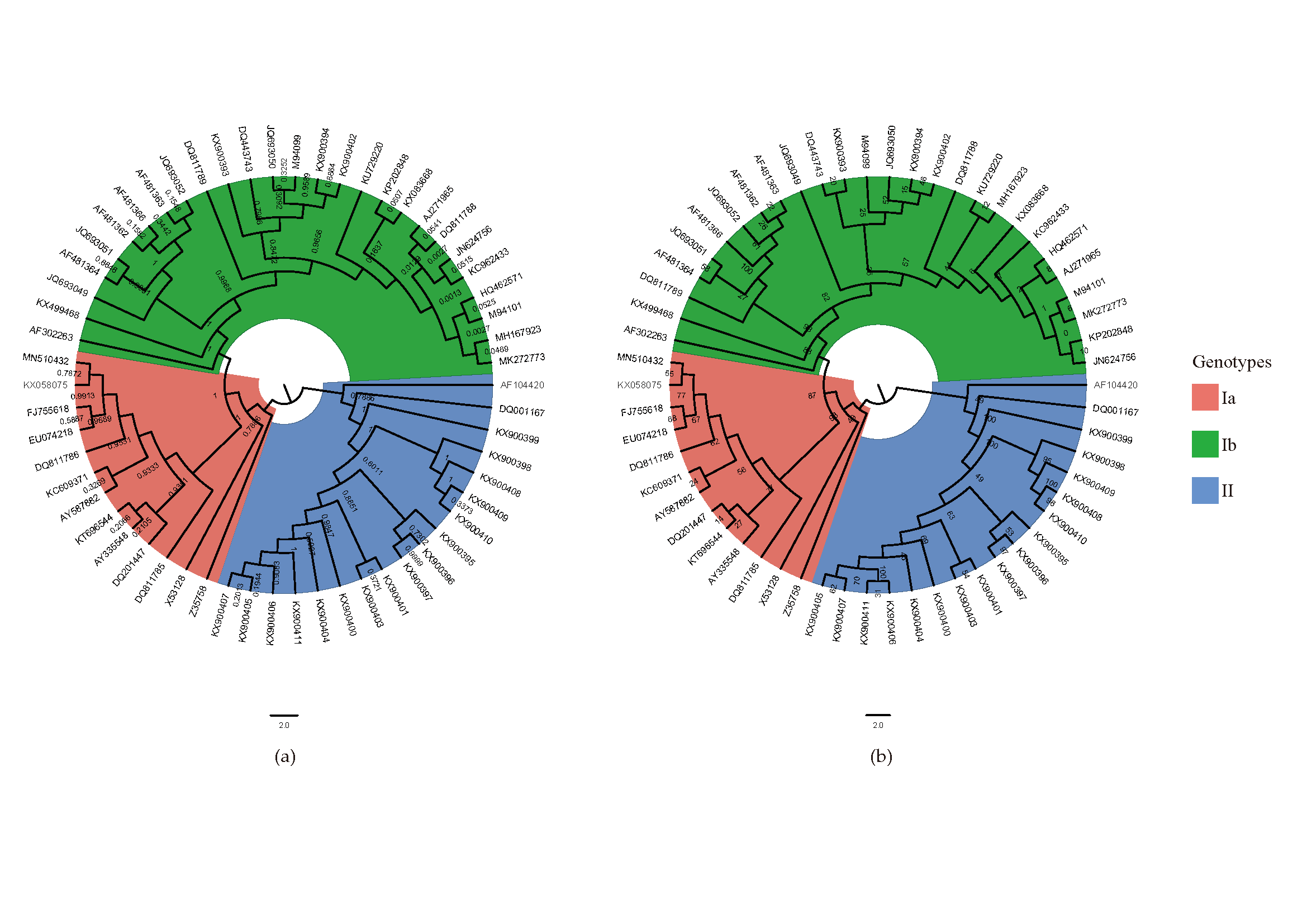
**

**Figure S2.** Phylogenetic trees of the partial *spike* gene of TGEV. (**a**) Bayesian inference tree of the partial *spike* gene of TGEV. Posterior probability values calculated by MrBayes are shown at each node. (**b**) Maximum likelihood tree of the partial *spike* gene of TGEV. Bootstrap support values computed by RAxML are indicated on the nodes of tree. Scale bar at the bottom of the figure indicates a length corresponding to 2.0 nucleotide substitutions per site. The colored sectors represent three genotypes of TGEV.

**
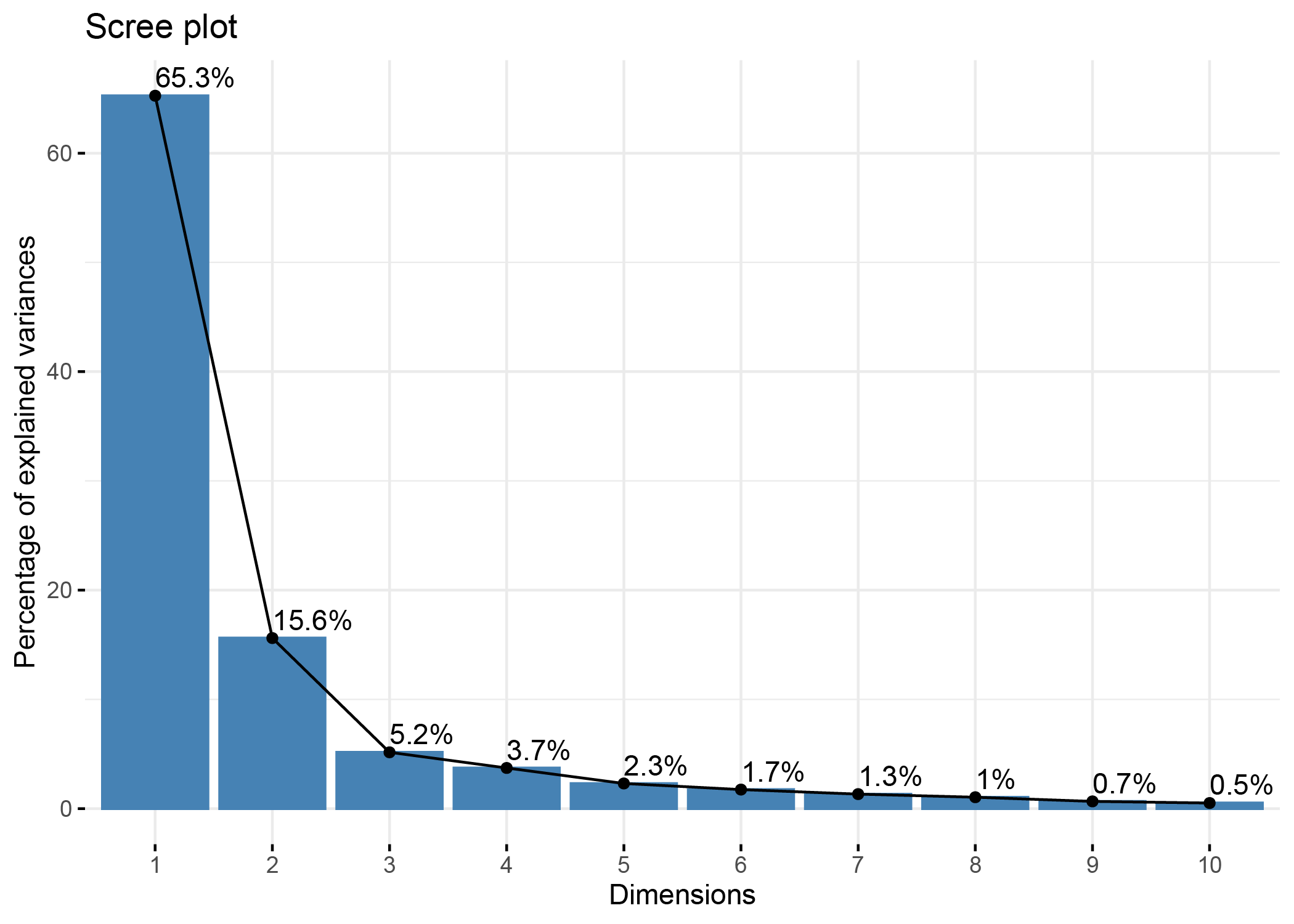
**

**Figure S3.** PCA scree plot based on the RSCU values of TGEV complete CDS. Scree plot extracts and visualizes eigenvalues (variances) from the RSCU values, and shows the proportion of total variance for each principal component (dimension) in descending order of magnitude.


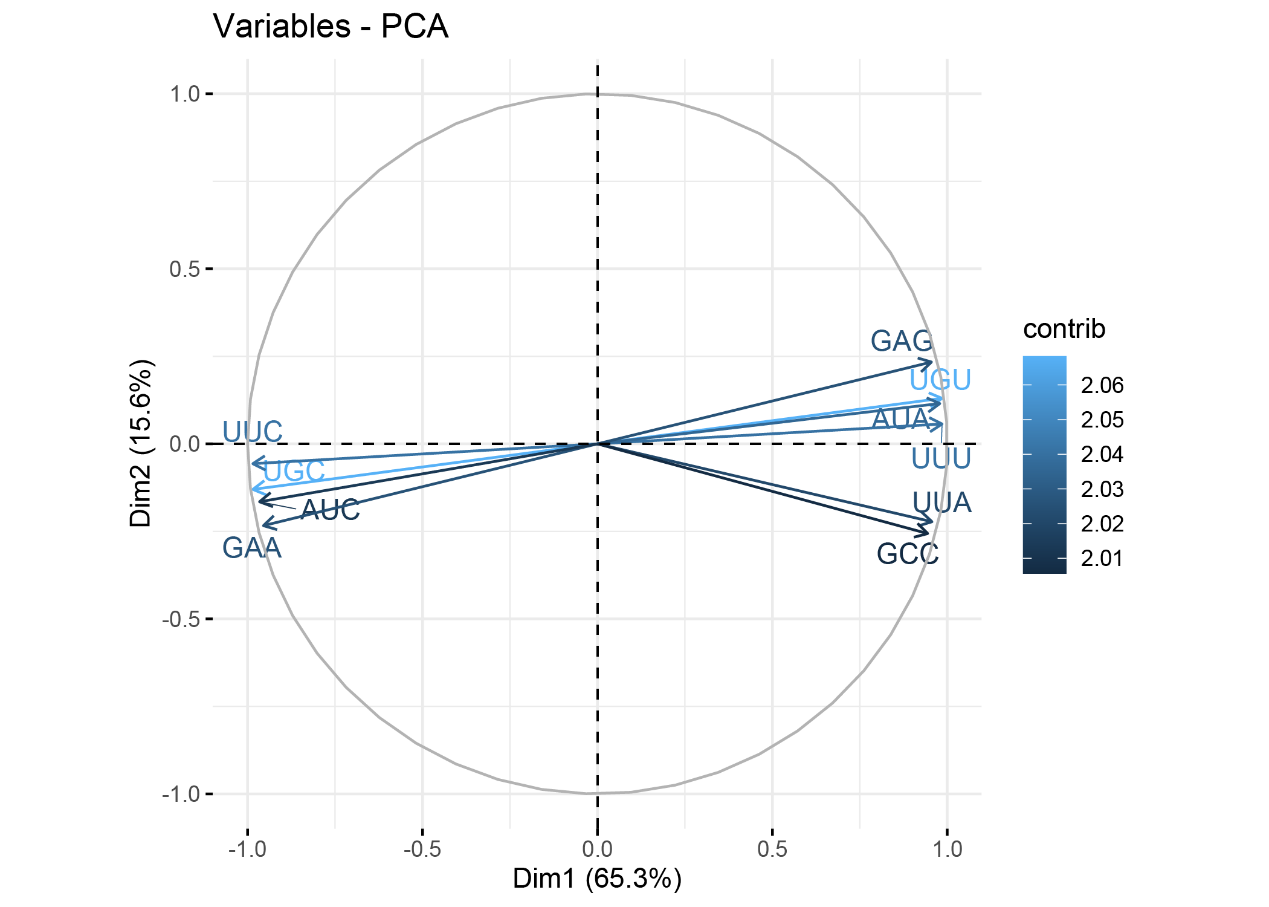


**Figure S4.** The variable correlation plots of PCA. The correlation circle with scaled coordinates of the variables' projection are represent. The top 10 variables and its directions are represented.
